# Genome-wide landscape of miRNA-mRNA-lncRNA-circRNA ceRNA network in Nanos2 deficient mice

**DOI:** 10.1371/journal.pone.0325260

**Published:** 2025-06-27

**Authors:** Hongyi Li, Yuan Li, Yuting Wang, Lijian Liu, Ji Cui, Mao Zhang, Yiming Yan

**Affiliations:** College of Biology and Agriculture, Shaoguan University, Guangdong, China; University of Helsinki: Helsingin Yliopisto, FINLAND

## Abstract

*Nanos2* plays a key role in self-renewing spermatogenic stem cells (SSCs) and maintains the stem cell state during spermatogenesis. Alleles of the *Nanos2* gene knockout showed germline ablated but otherwise structurally normal. To identify the probable ceRNA regulator involved in the process of spermatogenesis by *Nanos2*, whole transcriptome sequencing was performed in the testes between *Nanos2* knock out mice and wild type mice. Finally, a total of 8644 Differentially expressed (DE) mRNAs,180 DE miRNAs, 9538 DE lncRNA and 481 DE circRNAs were identified. Three of each RNAs were selected randomly and identified by real-time PCR to verify the accuracy of sequencing. GO and KEGG functional enrichment analyses revealed similar result of DE mRNAs and target of DE miRNAs/lncRNAs/ circRNAs, mainly involved in the generation, composition, and activity of sperm cells. Furthermore, the regulatory ceRNA network of miRNA(up)-circRNA-lncRNA-mRNA and miRNA(down)-circRNA-lncRNA-mRNA were constructed based on the common targeted miRNA.The results enable us to better understand the interaction of coding RNA and non coding RNA in regulating the generation of spermatogenic stem cells through *Nanos2* pathway, and also provided novel insights into molecular mechanism of spermatogenesis.

## Introduction

Transcriptomic analyses have revealed that only approximately 2% of the transcriptome encodes proteins [1]. The majority of transcripts are noncoding RNAs (ncRNAs), including microRNAs (miRNAs), long ncRNAs (lncRNAs), and circular RNAs (circRNAs), which play significant role in great progress of cellular life [2,3]. These ncRNAs have been showed to be critically involved in the reproductive system, where they participate in the regulation of essential events during spermatogenesis, including key molecular expression, cell differentiation, sperm morph-cellular maturation and sperm competitiveness [4–6].

Recent studies have shown that lncRNAs and circRNAs can act as miRNA sponges and compete for a limited pool of miRNA, forming “competitive endogenous RNA” (ceRNA) to control the subsequent post-transcriptional regulation [7]. circRNA-lncRNAs-miRNA-mRNA ceRNA regulatory network mediated the effects of testicular heat exposure [8].Genome-wide identification of mRNAs, lncRNAs, circRNAs and miRNAs can take significant effect in the molecular mechanisms of mouse testis development [9].

In mammals, The sustained sperm production required for male fertility is based on spermatogenic stem cells (SSCs), a subset of the undifferentiated spermatogonial population. SSCs are essential for maintaining lifelong spermatogenesis [10], as they posses the capacity to self-renew, ensuring a sufficient pool of stem cells, and to differentiate into daughter cells that ultimately give rise to spermatozoa [11].Multiple factors have been reported to be associated with self-renewal and differentiation of SSCs. The extrinsic cell factor *GDNF* can activates multiple downstream pathways by binding to its receptors *RET* and *GFRα1*, which plays the central role for regulation of fate determination of SSCs [12].The transcription factor *ETV5*, expressed in both Sertoli and germ cells [13,14], collaborates with *GDNF* to mediate the expression of genes critical for SSCs self-renewal [15]. Additionally, *Nanos2*, a zinc-finger RNA-binding protein, is a key regulator of SSCs self-renewal and stem cell state maintenance during spermatogenesis [16]. Knockout studies in male mice, pigs, cattle and goats have demonstrated that the absence of *Nanos2* results in germline ablation while leaving somatic structures intact [17], Conversely, over-expression of *Nanos2* leads to reduced proliferation and the excessive accumulation of SSCs [16].

In this study, we constructed a model of testicular *Nanos2* deficient mice, testes of three knockout mice and three control mice were used for whole transcriptome sequencing. Differentially expressed lncRNAs, circRNAs, miRNAs, and mRNAs identified from the whole transcriptome data will be used to construct RNA regulatory networks (lncRNA-miRNA-mRNA- lncRNA/circRNA) relevant to spermatogenesis. It is expected to find out which networks were involved in the generation and regulation of SSCs through the *Nanos2* deficient model.

## Materials and methods

### Animals and sample preparation

The heterozygous *Nanos2* (*Nanos2*^+/-^) C57BL/6J mouse model was generated by Cyagen Biosciences, using the CRISPR/Cas9 gene-editing system. F0 founder animals were identified by PCR followed by sequence analysis, and the *Nanos2*^+/-^ mice were selected to bred with wild-type mice (*Nanos2*^+/+^, WT) to confirm germline transmission and generate F1 offspring. Selected *Nanos2*^+/-^ mice (male and female) from F1 mice for mating and breeding F2 *Nanos2* (*Nanos2*^-/-^, KO) mice. All WT mice used in this experiment were litter mates of the KO mice. KO male mice with the same age were euthanized by the injection of sodium pentobarbital solution and testicular tissues were collected. The experimental procedures were approved by the Science and Technology Ethics Committee of Shaoguan University. The euthanasia procedure was meticulously performed to minimize animal distress, followed by immediate tissue collection in strict compliance with ethical guidelines and animal welfare considerations. All the mice were housed in ventilated independent cages under a 12-h/12-h light/dark cyclic environment and provided food and water ad libitum.

### Histopathological analysis

Testes were enucleated from three KO and three WT mice respectively and fixed in testicular tissue fixative (Servicebio), embedded in paraffin, sectioned, and stained with HE. Finally dehydrated and seal with neutral gum. HE stained sections were imaged on a NIKON ECLIPSE E100 microscope equipped with an NIKON DS-U3 Imaging system. The diameter of seminiferous tubules was measured by IPP6 software (Media Cybernetics). For immunohistochemistry detection, the paraffin sections were dewaxed, rehydrated, and incubated with mouse monoclonal anti-UCHL-1 antibody (Proteintech, 1:500 dilution). After incubated with Horseradish peroxidase (HRP) polymer conjugated secondary antibody (Servicebio, 1:200), the sections were washed and the staining signals were visualized using diaminobenzidine (DAB) and nuclei were counterstained with hematoxylin.

### Library Preparation and whole transcriptome sequencing

Total RNA was extracted from testes of three KO mice and three WT mice using Trizol reagent (Invitrogen, Carlsbad, CA, USA) according to the manufacturer’s procedure. A total amount of 2 μg RNA per sample was used for sequencing library construction. For mRNA, lncRNA, and circRNA analysis, the NEBNext^®^ Ultra^TM^ RNA Library Prep Kit for Illumina^®^ (NEB, Ipswich, MA, USA) was used to generate the Strand-specific libraries. library quality was assessed using the Agilent 5400 system and quantified by QPCR. Qualified libraries were sequenced on Illumina platforms with PE150 strategy in Novogene Bioinformatics Technology Co., Ltd (Beijing, China). For miRNA analysis, libraries were generated using the NEBNext^®^ Multiplex Small RNA Library Prep Set for Illumina^®^ (NEB, USA). The libraries were quantified using Qubit and qRT-PCR, and their size distribution was verified using a bio-analyzer. Finally, the quantified miRNA libraries were sequenced on an Illumina Novaseq^TM^6000 platform (Illumina, USA).

### Sequencing data analysis

Sequencing data was analyzed by Novogene Bioinformatics Technology Co., Ltd (Beijing, China). Sequencing raw reads were filtered using Perl 6 to remove reads with adapters and low-quality reads, with Q20/Q30 error rates (1%/0.1% error probabilities) were assessed using Illumina Casava (v1.8). Then the clean reads were aligned with the reference genome to obtain the localization information using HISAT2 (version 2.0.5) [18], and the mapped read counts were calculated using StringTie (v2.2.0) [19]. MRNAs were identified based on annotation information from the mus genome and transcriptome data. For lncRNA identification, transcripts longer than 200 bp and containing at least two exons were retained and compared with the known lncRNA database using gffcompare software. Then coding potential of these transcripts was further evaluated using Coding-Non-Coding-Index (CNCI) [20], Coding Potential Calculator (CPC) [21] and Pfam [22] tools. Transcripts lacking coding potential were considered as novel lncRNAs. CircRNAs were identified by find_circ [23] and CIRI2 [24]. For miRNA analysis, clean reads were aligned to miRBase (v22.0) to identify known miRNAs, and novel miRNAs were predicted using an integrated approach combining mirdeep2 [25] and miREvo [26].The Principal Component Analysis (PCA) of the samples were performed using DESeq2 software, and the dispersion for DESeq2 was estimated concurrently.

### Identification of differentially expressed (DE) RNA genes

The DESeq2 software package was used to identify DE lncRNAs, mRNAs, circRNAs and miRNA between KO and WT group [27]. The expression abundance of lncRNA and mRNA were calculated by fragments per kilobase per million mapped fragments (FPKM), the circRNA and miRNA expression levels were calculated and normalized to transcripts per million (TPM). The P-values was adjusted using the Benjamini & Hochberg method. Corrected P-value (padj) of 0.05 was set as the threshold for significantly differential expression by default. Genes with |log_2_(fold change)| > 1 together with padj-value < 0.05 were defined as significant differentially expressed. The heat-map R package was used to generate heat-maps of top 20 up/down DE genes base on the fold change.

### qRT-PCR analysis

Total RNA was extracted from testis tissues using Trizol (invitrogen). Reverse transcription was performed with a PrimeScript RT reagent kit with gDNA Eraser (TaKaRa, Japan) according to the manufacturer's instructions. The resulting cDNA from different samples were amplified using a CFX96 instrument (BioRad) with TB Green^®^ *Premix Ex Taq*^™^ II (TaKaRa, Japan), adhering to the manufacturers instruction. The PCR cycling conditions were as follows:1 cycle of 95 °C for 5 min, and 40 cycles of 95 °C for 15 s, 60 °C for 30 s,followed by a final melting curve analysis stage. The expression levels of the lncRNAs, circRNAs and mRNAs were normalized to the housekeeping gene β-actin, while miRNA expression levels were normalized to U6. Relative expression changes were calculated using the 2^−ΔΔCt^ method. The primer sequences were listed in Supplementary Table S1.

### GO and KEGG functional enrichment analysis

Gene Ontology (GO) enrichment of differentially expressed genes/target genes (padj<0.05) were analyzed using the Gene Ontology database (http://www.geneontology.org/), with padj<0.05 as significant enrichment. GO terms of the biological process (BP), molecular function (MF), and cellular component (CC) were presented base on the corrected P value. Kyoto Encyclopedia of Genes and Genomes (KEGG) pathway analysis were carried out using KOBAS [28]. Pathways of DE mRNA and DE miRNA were selected with padj<0.05 as significant enrichment. For the pathway analysis of DE lncRNA and DE circRNA, no significant results were obtained using a padj < 0.05 threshold, so P < 0.05 was applied to identify significant pathways. Pathways were classified and summarized according to information of KEGG PATHWAY Database (https://www.kegg.jp/kegg/pathway.html).

### Construction of interaction network

The targets of lncRNAs were identified using cis-acting (co-location) and trans-acting (co-expression) target gene prediction [29]. The targets of miRNAs were identified using the intersection result of miRanda and RNAhybrid. Additionally, targeted lncRNAs and cicrRNAs for DE miRNAs were predicted using miRanda software. Based on the ceRNA theory, lncRNAs/ circRNAs target gene pairs shared with the same miRNA binding site were identified to constructed ceRNA regulatory network with lncRNAs/circRNAs as decoy, miRNA as core, and mRNA as target. The regulatory networks of lncRNAs/circRNAs-miRNAs-mRNAs were visualized by Cytoscape.

### Statistical analysis

Data were plotted and statistically analyzed using GraphPad Prism 8 (GraphPad, San Diego, CA). Unpaired two-tailed Student’s t-tests were used to determine the statistical difference between two groups, and the data were presented as mean ± SEM.The number of animals tested (*n*) is indicated in the figure legends. In all statistical comparisons, a P value of <0.05 was considered statistically significant.

## Results

### Histological characteristics of testes in *Nanos2* KO mice

**Fig 1:**
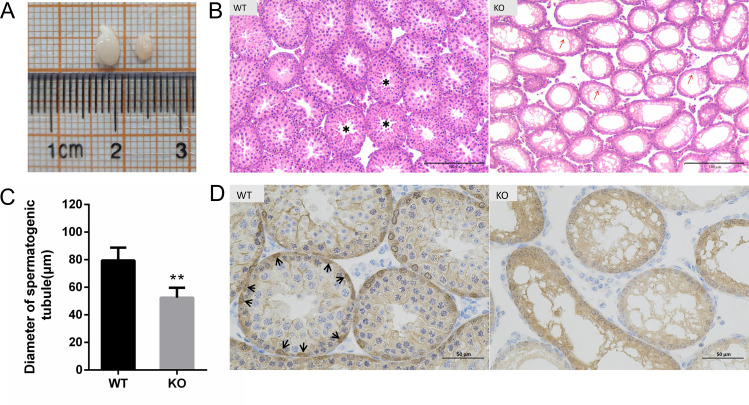
Histological analysis of the testes. (A) Representative images of testes from KO and WT mice. (B) Hematoxylin-eosin (HE) staining of testes cross-sections from KO and WT mice. KO represent the *Nanos2* knockout mice (*Nanos2*^-/-^) with smaller empty seminiferous tubules(red arrow), WT represent the wild-type mice (*Nanos2*^+/+^) with normal seminiferous tubules (marked with an asterisk), bar = 100μm. (C) Diameter of the seminiferous tubules. n_(WT)_=3, n_(KO)_=3, spermatogenic tubule no._(WT)_=79, spermatogenic tubule no._(KO)_=214. **,*P* < 0.01. (D)Immunohistochemistry analysis with UCHL1 antibody. The black arrows indicate UCHL1-positive SSCs, bar = 50μm.

Testes of *Nanos2* KO mice and WT mice with the same age (one month old) were collected for HE staining and immunohistochemistry detection. It can be clearly observed that the testis of *Nanos2* KO mice is significantly smaller than the WT mice (Fig 1A). HE staining of WT mice showed that the seminiferous tubules display a normal hierarchical structure, all germ cells are evenly arranged in the seminiferous tubule, while in *Nanos2* KO mice, these tubules were devoid of any germ cells (Fig 1B). Quantitative analysis of seminiferous tubule diameter demonstrated that the KO group exhibited significantly smaller tubule diameters than the WT group (*P* < 0.01,Fig 1C).To further determine whether germ cells, particularly SSCs, were still present in KO mice, we performed immunohistochemistry using an anti-UCHL1 antibody. The results showed a complete loss of spermatogonial stem cells and germ cells in the seminiferous tubules of KO testes(Fig 1D).Therefore, the reduced seminiferous tubule diameter in KO mice may results from germ cell loss.

### Overview of whole transcriptome sequencing

**Fig 2:**
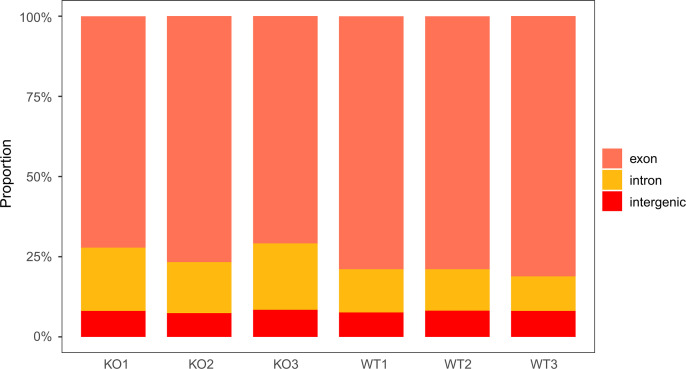
Proportion of mapping reads in each sample.

To identify the interaction network regulating spermatogenesis and testicular size, deep sequencing was performed on the testes of two groups of mice. After filtering out the low-quality reads and mapping to the reference genome, an average number of 93227699 clean reads with Q30 exceeding 92.48% were obtained for the identification of mRNAs, circRNAs and lncRNAs. An average number of 11585335 clean reads with Q30 above 97.41% were acquired for miRNAs identification. Finally, a total of 19373 mRNAs, 4544 novel circRNAs, 45228 lncRNAs (44613 exiting and 615 novel) and 986 miRNAs(967 exiting and 19 novel) were identified from the six testicles. Pearson correlation between samples showed that the correlation coefficients within the same group were all greater than 0.96, with most exceeding 0.98, indicating good reproducibility among the samples (S1 Fig). Principal component analysis revealed a distinct separation between KO and WT groups along PC1 (93.25% of total variance), indicating significant transcriptomics differences between the two groups.. The high intra-group sample cohesion further confirms robust experimental reproducibility (S2 Fig). The DESeq2 dispersion plot showed that the dispersion trend line (red curve) decreases with increasing expression levels, with the blue points distributed around the red trend line, and the dispersion values for most genes fall between 0.01 and 10 (S3 Fig). According to the mapping results, the distribution of reads across the exon region, intron region, and intergenic region of the genome was calculated. It was observed that the proportion of exons decreased while the proportion of introns increased in the KO group (Fig 2)

### Differential expression analysis of mRNA/ miRNA/lncRNA/ circRNA

**Fig 3:**
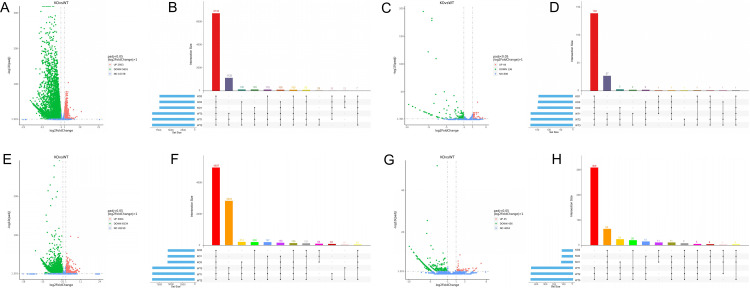
Expression patterns of mRNA, miRNA,lncRNA and circRNA between KO and WT mice. (A,C,E,G) Volcano plot of mRNA, miRNA, lncRNA and circRNA, red and green indicate up and down regulation, respectively. (B,D,F,H) Upset analysis of DE mRNA, miRNA, lncRNA and circRNA from each sample. Solid dots in the figure indicate presence in the sample. The number at the top of the bar represents the number of transcripts.

**Fig 4:**
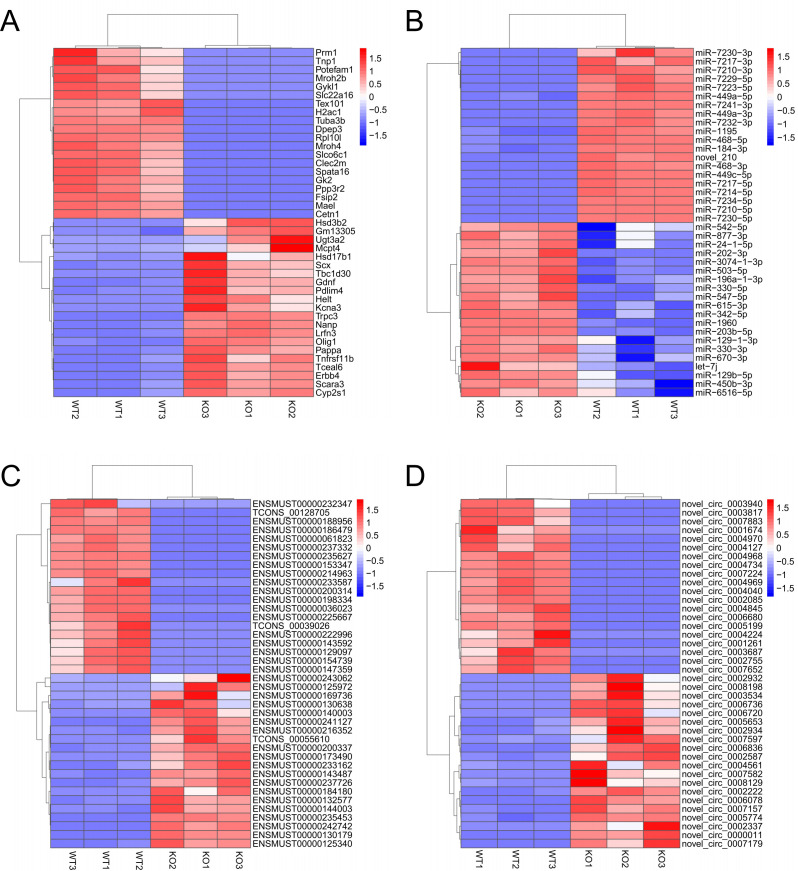
Clustered expression heatmaps of top 20 up and down expressed RNAs. (A-D) Hierarchical clustering heat-map of top 20 up and down expressed mRNA, miRNA, lncRNA and circRNA. Blue presents low expression, red presents high expression.

Compared with the WT mice, the KO mice showed the following differentially expressed genes: 8644 DE mRNAs, including 2953 genes up-regulated and 5691 down-regulated (Fig 3A), Among these, 6733 mRNAs expressed in both two group mice, while 12 and 1132 mRNAs were uniquely expressed in KO and WT mice, respectively(Fig 3B). 180 DE miRNAs were identified, with 44 up-regulated and 136 down-regulated (Fig 3C), among which 139 miRNAs were expressed in all samples and 27 miRNAs were exclusively expressed in WT testes (Fig 3D). 9538 DE lncRNAs were detected, comprising 3004 up-regulated and 6534 down-regulated (Fig 3E). Of these, 4937 lncRNAs expressed both in KO and WT mice, while 95 and 2,823 lncRNAs were uniquely expressed in KO and WT mice, respectively(Fig 3F). 481 DE circRNAs were identified, with 45 up-regulated and 436 down-regulated(Fig 3G), of which 65 circRNAs were expressed in all samples, and 11 and 309 circRNAs uniquely expressed in KO and WT mice, respectively (Fig 3H). The top 20 up-regulated and down-regulated genes for each RNA type were clustered and visualized in heat-maps (Figs 4A-D).

### qRT–PCR verification of DE mRNA/ miRNA/lncRNA/ circRNA

**Fig 5:**
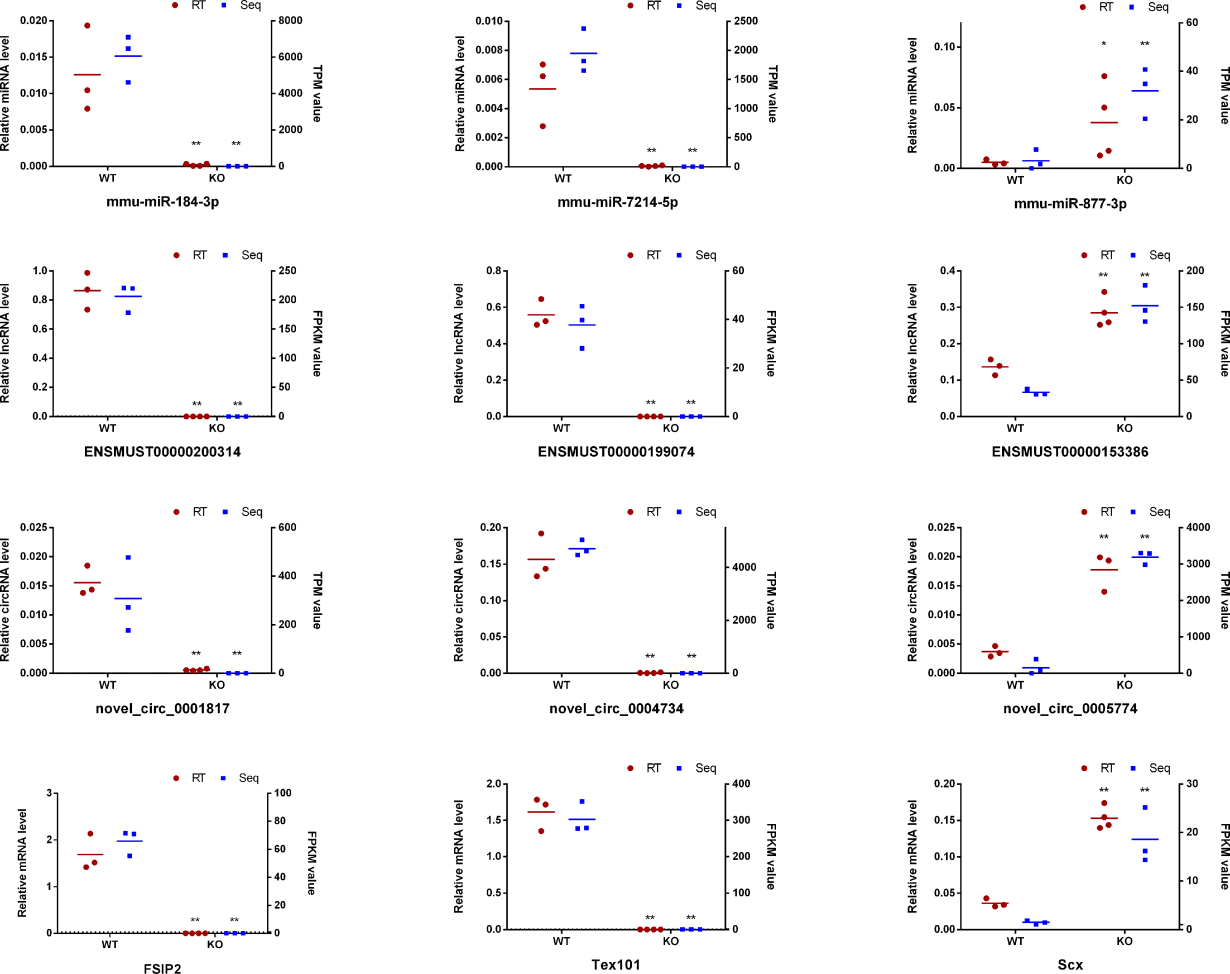
Validation of RNA sequencing data by RT-qPCR. The left y-axis represents the RT-qPCR result, miRNA data were normalized to the level of U6, the expression levels of lncRNAs, circRNAs and mRNAs were normalized to β-actin. The right y-axis displays the adjusted RNA-seq value. n_(KO)_=4,n_(wt)_=3.

To further verify the sequencing results of whole transcriptome, 3 DE miRNAs (mmu-miR- 184-3p, mmu-miR-7214-5p, mmu-miR-877-3p), 3 DE lncRNAs (ENSMUST00000200314, ENSMUST00000199074, ENSMUST00000153386), 3 DE circRNAs (novel_circ_0005774, novel_circ_0001817, novel_circ_0004734) and 3 DE mRNA (*Tex101*, *Dkkl1*, *Scx*) were randomly selected for expression analysis using RT-qPCR. The qPCR result showed that the expression patterns of the selected RNAs were highly consistent with the transcriptome sequencing data (Fig 5), confirming the reliability and accuracy of the sequencing results for subsequent analysis.

### Functional enrichment analysis of DE RNAs

**Fig 6:**
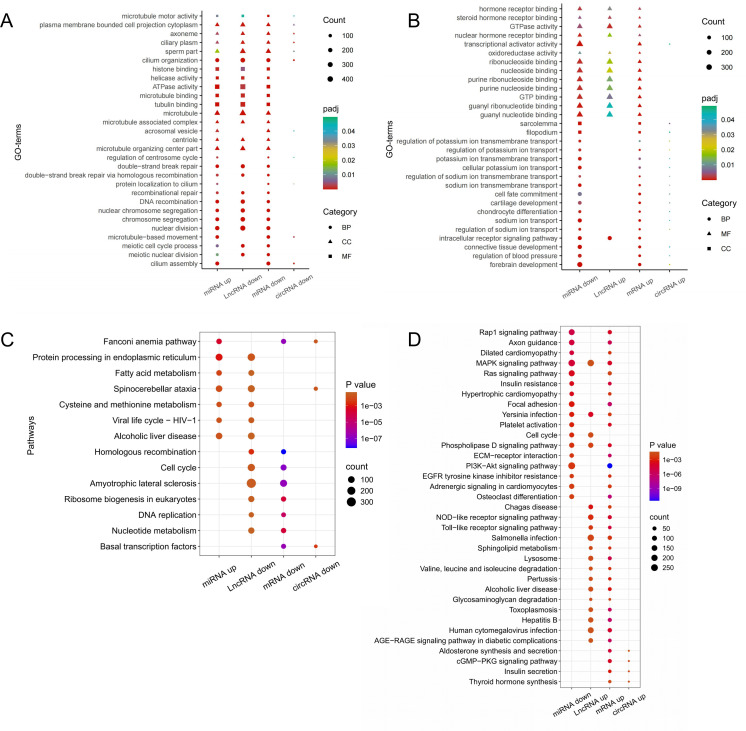
GO functional annotation and KEGG enrichment analysis of DE mRNAs and targets of DE miRNAs/lncRNA/ circRNA. (A) Common GO terms of up-regulated miRNAs and down-regulated mRNAs-lncRNAs/circRNAs. (B) Common GO terms of down-regulated miRNAs and up-regulated mRNAs-lncRNAs/circRNAs. Circular plots represent biological process (BP), Square plots represent cellular component (CC), triangle plots represent molecular function (MF). (C) Common pathways of up-regulated miRNAs and down-regulated mRNAs-lncRNAs/circRNAs. (D) Common pathways of down-regulated miRNAs and up-regulated mRNAs-lncRNAs/ circRNAs.

GO classifications of DE mRNAs revealed similar results with targets of DE miRNAs, DE lncRNAs and DE circRNAs. Targets of up-regulated miRNAs shared common GO terms with down-regulated mRNAs-lncRNAs/circRNAs, particularly those related to Spermatogenesis. Specifically, GO terms of miRNAs(up)-mRNAs-lncRNAs were mainly focused on ATPase activity, microtubule binding, protein localization to cilium, meiotic cell cycle process and so on. GO terms for miRNAs(up)-mRNAs-circRNAs were enriched in 11 categories, including acrosomal vesicle, protein localization to cilium and cilium assembly. Notably, 6 GO terms, such as sperm part, were enriched in both miRNAs(up)-mRNAs-lncRNAs and miRNAs(up)-mRNAs- circRNAs(Fig 6A). Similarly, down-regulated miRNAs shared the same GO terms with up-regulated mRNAs-lncRNAs/circRNAs, most of which were related to nucleoside binding and ion transport (Fig 6B).

KEGG pathway enrichment of DE miRNAs, DE mRNAs, DE lncRNAs and DE circRNAs were analyzed and pathway that intersected with other RNAs were selected. The integrated result showed that only Spinocerebellar ataxia pathway was enriched in the intersection of miRNAs (up)-mRNAs-lncRNAs, and only Fanconi anemia pathway enriched in the intersection of miRNAs (up)- mRNAs-circRNAs (Fig 6C). For miRNAs (down) pairs, MAPK signaling pathway, Yersinia infection and Phospholipase D signaling pathway were enriched in the intersection of miRNAs (down)-mRNAs-circRNAs whereas no pathways were shared in the intersection of miRNAs (down)-mRNAs- lncRNAs (Fig 6D).

### Construction of ceRNA regulatory network

**Fig 7:**
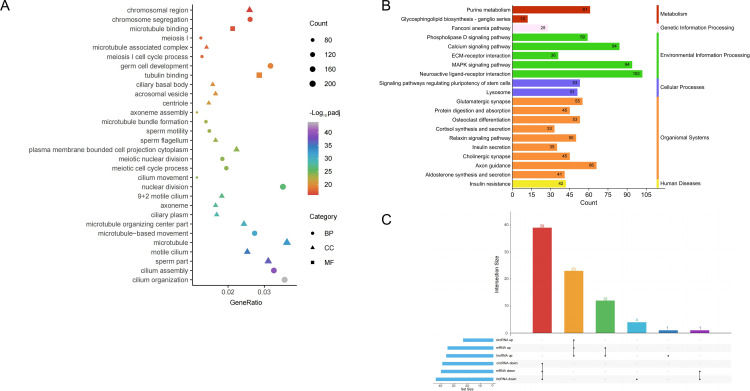
Analysis of miRNA-mRNA-circRNA-lncRNA ceRNA. (A) The top 30 GO therms enriched with co-expression mRNA of DE miRNA-circRNA-lncRNA. (B) The top 20 KEGG pathways enriched with co-expression mRNA of DE miRNA-circRNA-lncRNA. (C) Upset analysis of common miRNAs that targeted with mRNA, circRNA and lncRNA.

To further elucidate the interplay of miRNAs, mRNAs, lncRNAs and EcircRNAs, interacting DE pairs of circRNA-miRNA, mRNA-miRNA, and lncRNA-miRNA were predicted. Co-expression pairs of circRNA/lncRNA-miRNA and mRNA-miRNA were interacted to construct miRNA-circRNA-mRNA and miRNA-lncRNA-mRNA regulatory networks (S1 and S2 Files). GO analysis of common mRNAs in these networks showed that target mRNAs were mainly involved in sperm generation, composition, and activity (Fig 7A). KEGG pathway enrichment analysis identified the 20 most significantly enriched pathways associated with these common mRNAs, which were further categorized into six major groups. The networks predominantly focused on the environmental information processing and Organismal Systems (Fig 7B).

**Fig 8:**
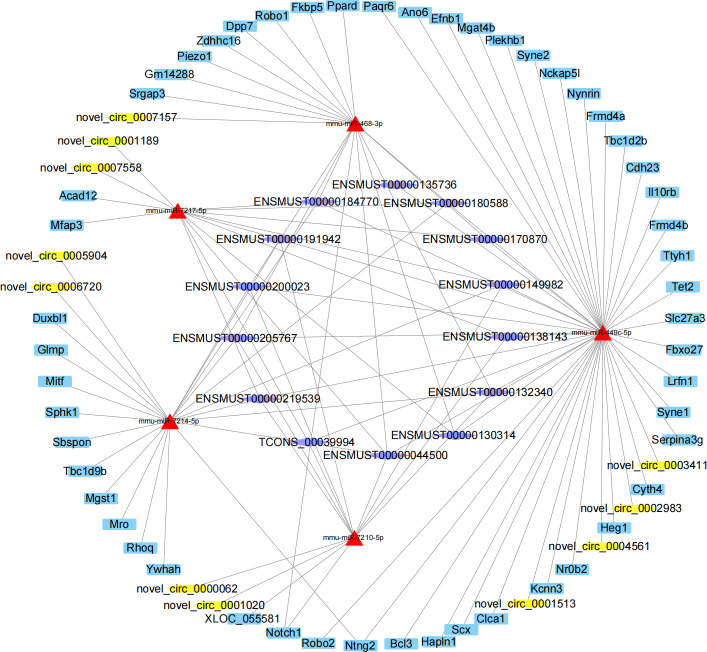
The interaction network of up regulated miRNA with down regulated mRNA, circRNA and lncRNA. Hexagon nodes represent lncRNAs, triangle nodes represent miRNAs, diamond nodes represent circRNAs and round rectangle nodes represent mRNAs/genes.

**Fig 9:**
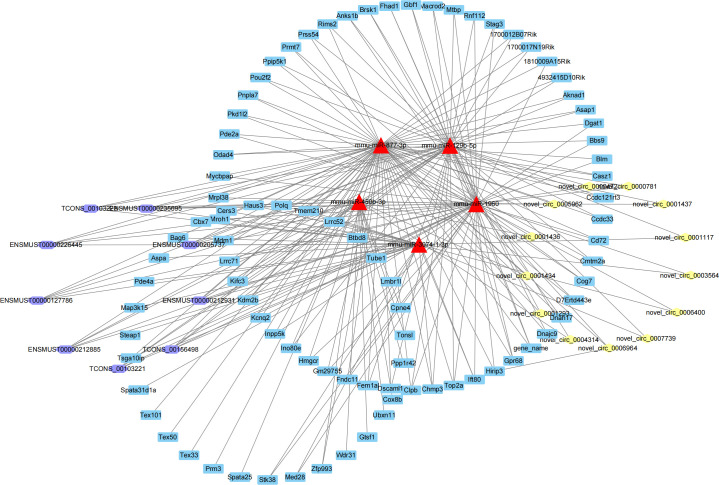
The interaction network of down regulated miRNA with up regulated mRNA, circRNA and lncRNA. Hexagon nodes represent lncRNAs, triangle nodes represent miRNAs, diamond nodes represent circRNAs and round rectangle nodes represent mRNAs/genes.

Moreover, the common targeted miRNAs of the networks were calculated, among which 39 down-regulated miRNAs exhibited binding relationships with up-regulated mRNA, lncRNA, and circRNA, while 23 up-regulated miRNAs targeted with the down-regulated mRNA, lncRNA, and circRNA (Fig 7C).Then, top 5 up-regulated miRNAs were selected from the 39 common miRNAs to construct a comprehensive miRNA(up)-circRNA-lncRNA-mRNA regulatory network(Fig 8). This network comprised 115 node, including 5 DE miRNAs, 13 DE circRNAs, 9 DE lncRNAs and 88 DE mRNAs. Similarly, top 5 down-regulated miRNAs from the 23 common miRNAs were used to constructed a ceRNA networks of miRNA(down)-circRNA-lncRNA-mRNA(Fig 9), which included 5 DE miRNAs, 11 DE circRNAs, 14 DE lncRNAs and 52 DE mRNAs.

## Discussion

The male specific RNA binding protein *Nanos2* plays a crucial role in inhibiting the cell cycle in germ cells, its expression starts in mitotic cells and induces mitotic arrest [30]. *Nanos2* maintains SSC populations by modulating mRNA stability and regulating key signaling pathways essential for self-renewal [31]. Conditional knockout of *Nanos2* gene in testes results in the progressive depletion of SSCs within seminiferous tubules, ultimately leading to the complete loss of both spermatogonia and mature sperm cells. This cellular depletion manifests phenotypically as a significant reduction in testicular size in *Nanos2* KO mice compared to their WT counterparts. In this study, we systematically characterized the co-expression patterns of lncRNAs, circRNAs, miRNAs, and mRNAs in the *Nanos2* deficient testis, which may provide new insights for the regulation of spermatogenesis.

Through whole transcriptome sequencing and genomic mapping analysis, we observed a significant shift in RNA composition in KO testis, characterized by a decreased proportion of exonic reads and a corresponding increase in intronic reads.. The intron-aligned reads likely originate from either precursor mRNA transcripts or introns retained by alternative splicing events, and the reads aligned to the intergenic region may come from ncRNA or a few DNA fragments [32].*Nanos2* Knockout causes the deficient of spermatogonia and sperm cells, which may in turn leads to the loss or down-regulation of genes related to their generation and composition, thereby reducing the proportion of exons.

To identify candidate RNAs that related to the depletion of spermatogonial stem cells caused by *Nanos2* deficiency,we conducted comprehensive transcriptomic profiling to identify DE lncRNAs/circRNAs/miRNAs/mRNAs between WT and KO testes. As revealed in the heat-map, most mRNAs that down-expressed in the KO testis were related to the generation and composition of sperm cells. Among these, FSIP2 emerged as a top candidate, encoding a structural component of the sperm fibrous sheath. This intra-flagellar transport protein plays crucial roles in axonemal assembly, mitochondrial selection and the termination of mitochondrial sheath extension during spermatogenesis [33]. *MAEL* gene was located in the mitochondria of ejaculated sperm, disruption of *MAEL* in male mice leads to the sterile phenotype of testes without postmeiotic germ cells [34].Conversely, up-regulated mRNAs in KO testes were predominantly associated with testicular Leydig cells and Sertoli Cell. Testosterone is mainly synthesized by testicular Leydig cells and *Hsd3b2* is a key enzyme in testosterone synthesis [35]. *Amh* is produced by Sertoli Cell in male and high concentrations of *Amh* promotes cell apoptosis [36].

GO classifications and KEGG enrichment of DE RNAs revealed that there was mutual correlation between DE mRNAs and targets of DE miRNAs/lncRNAs/circRNAs. In the result of miRNAs(up)-mRNAs-lncRNAs/circRNAs, 6 GO terms were enriched across all categories, with two-thirds predominantly associated with cellular component, indicating that these RNAs may interact with each other to regulate the composition of spermatogonia and sperm cells. In addition, Fanconi anemia(FA) pathway was enriched in the intersection of miRNAs(up)-mRNAs-circRNAs. FA pathway was mainly involved in DNA cross-linking damage repair and replication stress response, which was required for primordial germ cells development and normal fertility. Deficiency of FA pathway results in DNA damage and the proliferation defects of primordial germ cells ultimately impair fertility, with male mice showing testicular shrinkage and abnormal sperm production [37]. On the other hand, common GO terms of miRNAs(down)-mRNAs-lncRNAs were mainly enriched in MF (such as nucleoside binding), while miRNAs(down)-mRNAs-circRNAs were showed significant involvement in BP (such as ion transport). The development of germ cells require a steady supply of nutrients, including nucleosides, sugars, hormones and metabolites that do not readily diffuse across membranes, and the movement of these nutrients must involve transporters [38]. Consequently, the underdevelopment of germ cells may leads to the accumulation of nucleoside binding proteins and ion transporters. Meanwhile, the MAPK signaling pathway emerged as a key enriched pathway in the intersection of miRNAs (down)-mRNAs-lncRNAs. In testis, MAPK signaling modulates blood-testis barrier integrity through regulation of structural protein expression [39], indicating potential roles of these RNAs in the regulation of blood-testis barrier. The description above indicates that there are potential miRNA-mRNA-circRNA-lncRNA ceRNA interactions involved during spermatogenesis.

According to the ceRNA hypothesis, lncRNAs and circRNAs function as molecular sponges that compete with mRNAs for miRNA binding, thereby regulating specific target mRNAs. Based on this principle, we predicted miRNA-mRNA, miRNA-circRNA and miRNA-lncRNA target pairs and constructed ceRNA networks centered on shared miRNA. In these networks, core miRNAs act like mediators among lncRNAs, circRNAs and mRNAs. In the network of up regulated miRNAs, the top 5 miRNAs mmu-miR-129b-5p, mmu-miR-877-3p, mmu-miR-1960, mmu-miR-3074–1-3p and mmu-miR- 450b-3p target to the same 9 lncRNAs. Among them, genes co-location with lncRNA *Dnah8* and lncRNA *Topaz1* were associated with male infertility [40,41]. It has been reported that inhibition of miR-450b-3p expression can activate the mitochondrial apoptosis signaling pathway in spermatocytes [42], while mmu-miR-877-3p may implicated in fertilization processes [43]. The network also includes respiratory chain genes *Cox8b*, testis expressed gene *Tex101*,*Tex50* and *Tex33*, providing valuable insights into the regulatory mechanisms of spermatogenesis. Meanwhile, we also constructed the ceRNA network of top 5 down regulated miRNAs. One of the sub network was mmu-miR-449c-5p-lncRNAScarb1-Heg1/ Scx/Hapln1. lncRNA *Scarb1* originated from the sense chain of *Scarb1* gene, which was the specific marker of testicular Leydig cells [44]. Target gene *Heg1* was mainly expressed in endothelial cells [45], *Scx* was found to induce the later stages of Sertoli cell differentiation [46], and Hapln1could promote cell adhesion [47]. This sub-network may facilitate the proliferation of other cells such as Leydig cells, endothelial cells, and Sertoli cells in the absence of SSCs, and tightly bind these cells together to maintain the morphology of the testis. Which supported by the up regulation of *Sox9* and Fat1, the marker genes of Sertoli cells and calcium binding protein respectively.The circRNA identified in these networks are novel, and their functional roles in spermatogenesis require further experimental validation

Compared to existing studies on the role of noncoding RNA in spermatogenesis, multiple reports revealed that lncRNA *Gm2044* has regulatory effects on the processes of spermatogenesis. LncRNA *Gm2044* is highly expressed in pachytene spermatocytes and during meiosis in the mouse testis [48]. Its expression level decreases when the number of spermatocytes is reduced due to their differentiation into spermatids [49]. However, in cases of non-obstructive azoospermia with spermatogonia arrest, the expression of lncRNA *Gm2044* is significantly up-regulated [50]. lncRNA *Gm2044* also functions as a miR-335-3p sponge to increase the level of miR-335-3 p’s direct target protein, Sycp1 [51], which is essential for meiois [52]. In this study, spermatogonia and sperm cells were disappeared in the testis of KO mice, thereby sycp1 was down-regulated in the KO testis, but the expression of lncRNA *Gm2044* still significantly up-regulated, indicating that lncRNA *Gm2044* was not only expressed in the spermatocytes.

Furthermore, we classified the GO functional annotation of common mRNAs in the miRNA-circRNA-mRNA and miRNA-lncRNA-mRNA networks, with sperm part, germ cell development, cilium organization, sperm flagellum, meiotic cell cycle process, and sperm motility the most enriched terms, which associated with the generation, composition, and activity of sperm cell. This result was consistent with the result of HE staining, indicating that the networks we constructed maybe the potential regulator in spermatogenesis.

## Conclusion

In conclusion, whole-transcriptome sequencing technology was employed to reveal the expression profiles of miRNAs, mRNAs, lncRNAs and circRNAs in the testis of *Nanos2* deficient mice. Based on the data, we constructed ceRNA regulatory networks of miRNA(up)-circRNA- lncRNA-mRNA and miRNA(down)-circRNA-lncRNA-mRNA with top 5 common miRNAs as the core. These comprehensive genomic data enabled us to better understand the interaction of coding RNA and non coding RNA in regulating the generation of SSCs through *Nanos2* pathway, and also provided novel insights into molecular mechanism of spermatogenesis.

## Supporting information

S1 Fig Pearson correlation between samples. (TIF)

S2 Fig 3D principal component analysis (PCA) clustering visualization across all samples. The red squares represent the three KO samples, while the blue circles denote the three WT samples.
(TIF)

S3 Fig The DESeq2 dispersion plot of the smaples. Red curve represents DESeq2-fitted dispersion trend line (mean-dispersion relationship); blue points represent final shrunken dispersion values (used for statistical testing); black dots represent raw dispersion estimates for each gene.
(TIF)

S1 Table Primers of real-time PCR detection. (DOCX)

S1 File miRNA-circRNA-mRNA network. (XLS)

S2 File miRNA-lncRNA-mRNA network. (XLS)
