# Supplementary figures and images for: Genome-wide landscape of miRNA-mRNA-lncRNA-circRNA ceRNA network in Nanos2 deficient mice
Source: PLoS One. 2025 Jun 27;20(6):e0325260. doi: 10.1371/journal.pone.0325260 (PMC12204511; doi:10.1371/journal.pone.0325260)

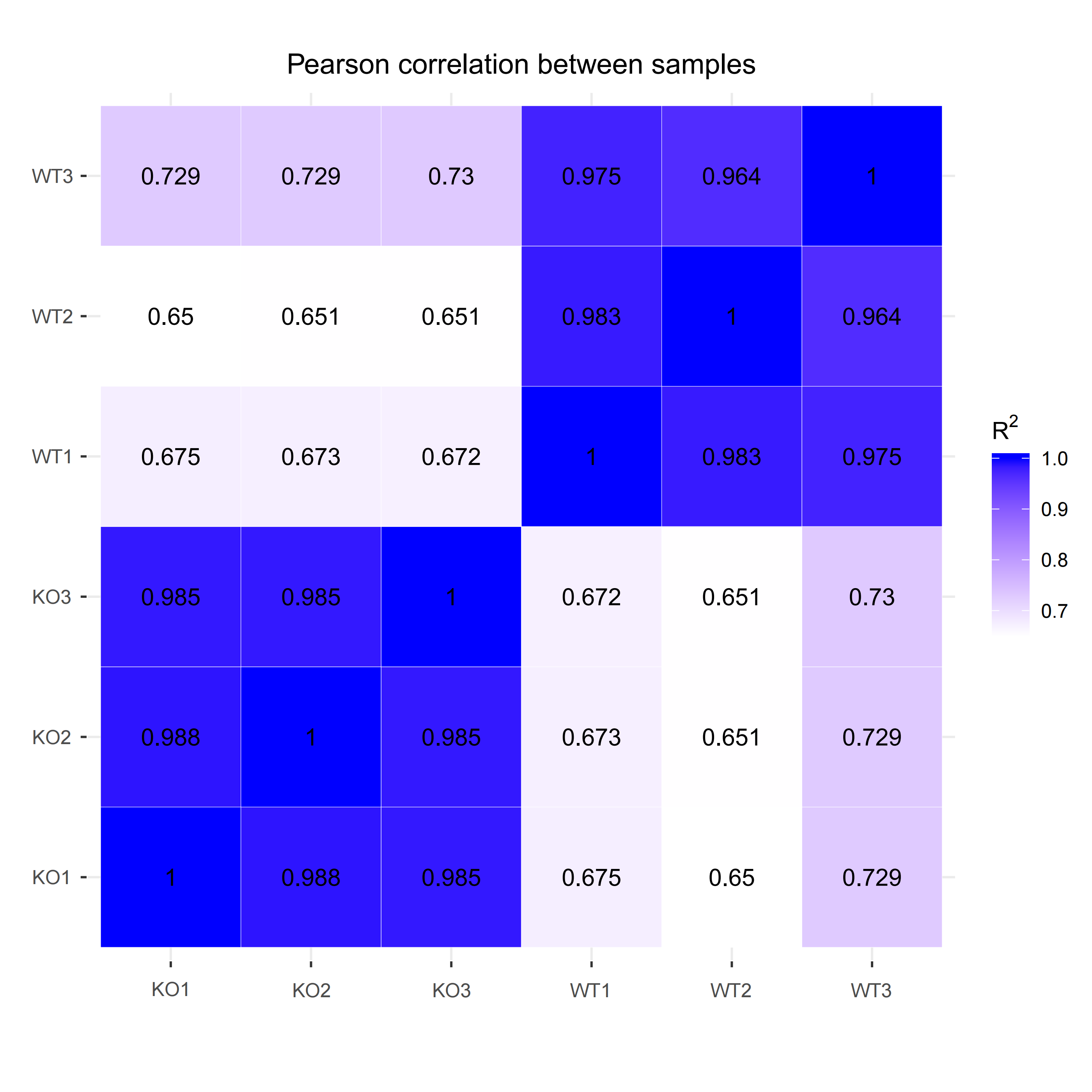

Supplement: S1 Fig — (TIF) [file pone.0325260.s001.tif]

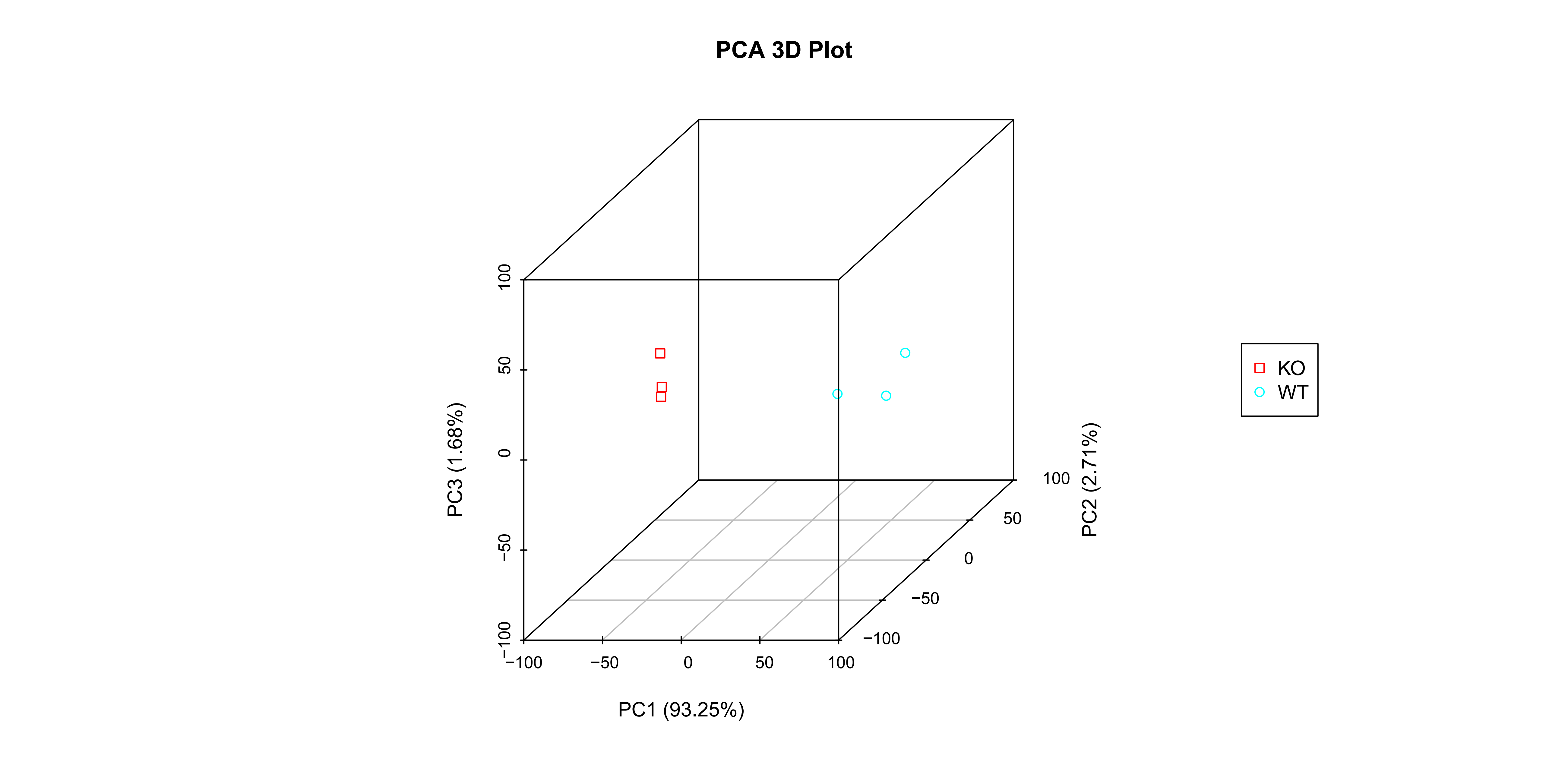

Supplement: S2 Fig — The red squares represent the three KO samples, while the blue circles denote the three WT samples. (TIF) [file pone.0325260.s002.tif]

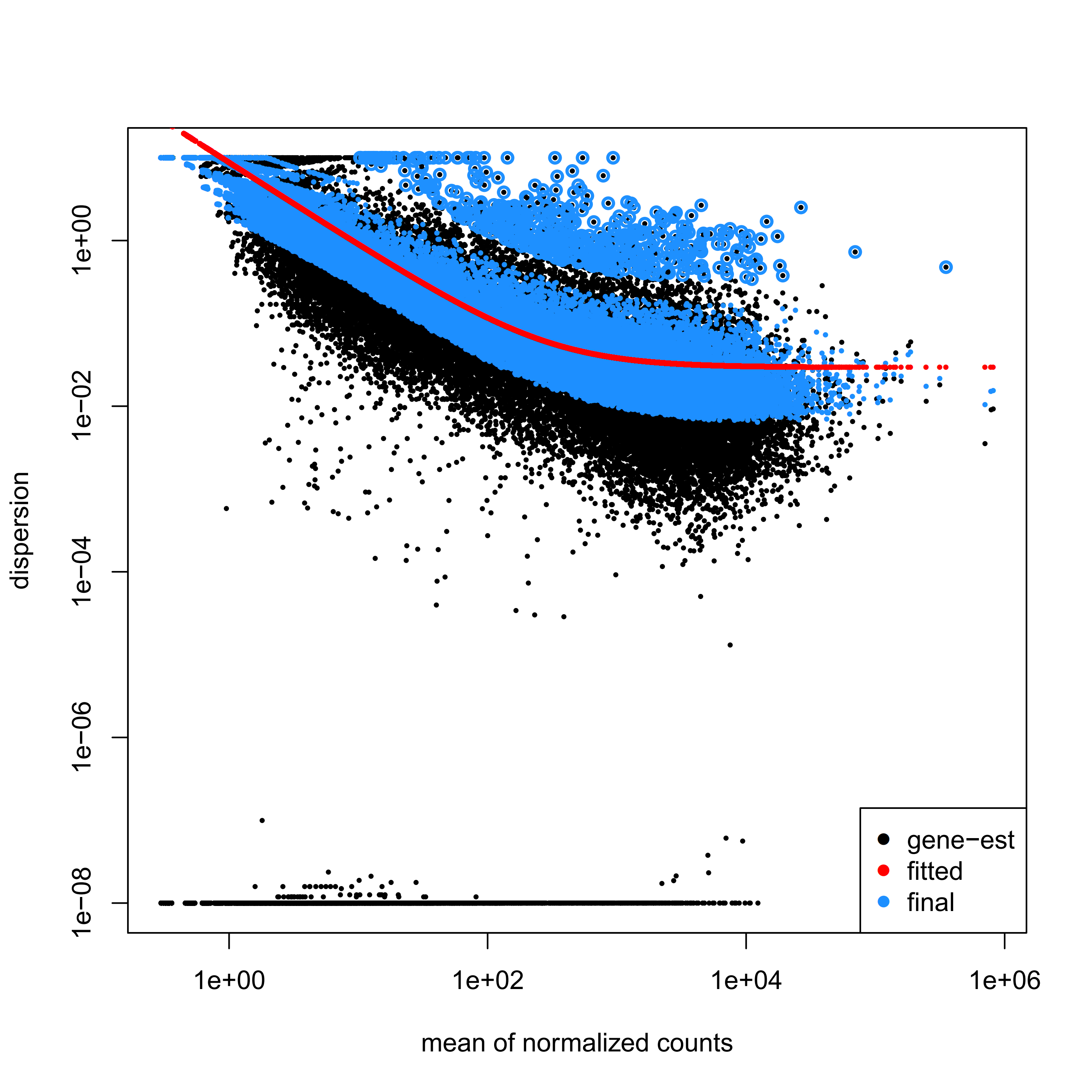

Supplement: S3 Fig — Red curve represents DESeq2-fitted dispersion trend line (mean-dispersion relationship); blue points represent final shrunken dispersion values (used for statistical testing); black dots represent raw dispersion estimates for each gene. (TIF) [file pone.0325260.s003.tif]
