# Supplementary material for: Genome-wide landscape of miRNA-mRNA-lncRNA-circRNA ceRNA network in Nanos2 deficient mice
Source: PLoS One. 2025 Jun 27;20(6):e0325260. doi: 10.1371/journal.pone.0325260 (PMC12204511; doi:10.1371/journal.pone.0325260)
Supplement: S1 Table — (DOCX) [file pone.0325260.s004.docx]

Table S1 Primers of real-time PCR detection

| Name | Sequence |
| --- | --- |
| mmu-miR-184-3p | GACGGAGAACTGATAAGGGT |
| mmu-miR-7214-5p | GCTTTCTGGGTTGGAATGAG |
| mmu-miR-877-3p | TGTCCTCTTCTCCCTCCTC |
| Universal Primers | GCTGTCAACGATACGCTACGT |
| Tex101-F | GGCGGTGACTTTCATCCAGTA |
| Tex101-R | ATGTTGGAGGTTGCTGTGGTC |
| Scx-F | TTTCTTCCACAGCGGTCGT |
| Scx-R | ATCGCCGTCTTTCTGTCACG |
| Dkkl1-F | TGTCGACTCTCAGCAGAACAC |
| Dkkl1-R | GGGTATGGTTGCCCATTCTG |
| LncRNA Rbakdn-F | AGCACCTCGGAAGTGAGAAG |
| LncRNA Rbakdn-R | GGATGGGCTGTTGGTCAGAG |
| LncRNA Myl10-F | CTCTTAACTCCTGCCCAGTTCA |
| LncRNA Myl10-R | TGAAGGCGTGCAGTATAGTCTC |
| LncRNA Maged1-F | GCTGGCATACTGGGAACGTA |
| LncRNA Maged1-R | TCTGCCCATGACCTCCTATG |
| circ_0001817 F | CCACGGTTTCTATTGAGCAT |
| circ_0001817 R | ATGTATCAAGCAAGCTCGTC |
| circ_0004734 F | CTTTATTCTCGTTCTCCTCG |
| circ_0004734 R | ATGTGACAGTACCTCCTCAT |
| circ_0005774 F | CTGCTCCCGTTCCATCCACA |
| circ_0005774 R | GGCGTGATGGGTGGGATGTT |
| U6 F | CTCGCTTCGGCAGCACATATACT |
| U6 R | ACGCTTCACGAATTTGCGTGTC |
| GAPDH F | CCAGGTTGTGTCCTGTGACT |
| GAPDH R | GCTTGACGAAGTGGTCGTTG |
